# FimH and Type 1 Pili Mediated Tumor Cell Cytotoxicity by Uropathogenic *Escherichia coli* In Vitro

**DOI:** 10.3390/pathogens12060751

**Published:** 2023-05-23

**Authors:** Shelly Roselyn Van Eyssen, Anastasia Samarkina, Ovgu Isbilen, Merve Suzan Zeden, Ender Volkan

**Affiliations:** 1 Biotechnology Research Center, Cyprus International University, Northern Cyprus, Mersin 10, 99258 Nicosia, Turkeyoisbilen@ciu.edu.tr (O.I.); merve.zeden@universityofgalway.ie (M.S.Z.); 2 Department of Pharmacy, Faculty of Pharmacy, Cyprus International University, Northern Cyprus, Mersin 10, 99258 Nicosia, Turkey; 3 Department of Microbiology, School of Biological and Chemical Sciences, University of Galway, H91TK33 Galway, Ireland

**Keywords:** UTI89, type 1 pili, fimbriae, cancer, MCF-7, MDA-MB-231, chaperone–usher, cytotoxicity

## Abstract

Uropathogenic *Escherichia coli* express hairlike proteinaceous surface projections, known as chaperone–usher pathway (CUP) pili. Type 1 pili are CUP pili with well-established pathogenic properties. The FimH adhesin subunit of type 1 pili plays a key role in the pathogenesis of urinary tract infections (UTIs) as it mediates the adhesion of the bacteria to urothelial cells of the bladder. In this study, two breast cancer cell lines, MDA-MB-231 and MCF-7, were used to demonstrate the cytotoxic activities of type 1 piliated uropathogenic *E. coli* UTI89 on breast cancer cells in a type 1 pili and FimH-mediated manner. *E. coli* were grown in static and shaking conditions to induce or inhibit optimal type 1 pili biogenesis, respectively. Deletion constructs of UTI89 Δ*fimH* and a complemented strain (UTI89 Δ*fimH*/p*fimH*) were further utilized to genetically assess the effect of type 1 pili and FimH on cancer cell viability. After incubation with the different strains, cytotoxicity was measured using trypan blue exclusion assays. UTI89 grown statically caused significant cytotoxicity in both breast cancer cell lines whereas cytotoxicity was reduced when the cells were incubated with bacteria grown under shaking conditions. The incubation of both MDA-MB-231 and MCF-7 with UTI89 Δ*fim* operon or Δ*fimH* showed a significant reduction in cytotoxicity exerted by the bacterial strains, revealing that type 1 pili expression was necessary for cytotoxicity. Complementing the Δ*fimH* strain with p*fimH* reversed the phenotype, leading to a significant increase in cytotoxicity. Incubating type 1 pili expressing bacteria with the competitive FimH inhibitor D-mannose before cancer cell treatment also led to a significant reduction in cytotoxicity on both MDA-MB-231 and MCF-7 cancer cells, compared to vehicle control or D-mannose alone, indicating the requirement for functional FimH for cytotoxicity. Overall, our results reveal that, as opposed to UTI89 lacking type 1 pili, type 1 piliated UTI89 causes significant cancer cell mortality in a FimH-mediated manner, that is decreased with D-mannose.

## 1. Introduction

Uropathogenic *Escherichia coli* (UPEC), a Gram-negative bacterium, is the primary causative agent of urinary tract infections (UTIs) [1,2]. Like many Gram-negative bacteria, UPEC also interacts with its environment using proteinaceous, filamentous, polymeric, nonflagellar fibers known as pili. The type 1 pili, assembled by the chaperone–usher pathway, mediates the bacterial attachment, biofilm formation and invasion of bladder urothelial cells [3,4,5,6,7,8]. In addition to *E. coli*, several other Gram-negative bacteria including *Klebsiella pneumoniae* and *Salmonella* spp. assemble type 1 pili [9].

Type 1 pili are composed of four different protein subunits (FimH, FimG, FimF and FimA), maintaining a noncovalent interaction to form the pilus fiber. The adhesin FimH is found at the tip of the fiber, situated there with two linker subunits FimG and FimF forming the short flexible fibrillar tip of the pilus attached to the rod, FimA. The pilus assembly is catalyzed by a periplasmic chaperone protein, FimC, and an outer membrane (OM) multidomain catalyst usher FimD [10,11]. The pilus subunits that are transferred into the periplasmic space via the sec machinery initially mediate a transient interaction with the chaperone of the system, FimC, which delivers the pseudofolded but stable subunits to the OM assembly platform, the usher (FimD) [11,12,13,14]. Using its multidomain structure, FimD catalyzes the assembly of the pilus where the chaperone–subunit complexes initially arrive at the N-terminus of the usher, uncorking the pore structure via mobilizing the plug domain and ultimate transfer to the C-terminal domains CTD1 and CTD2, followed by a protrusion through the pore of the usher [13]. Upcoming chaperone–usher subunits then follow the same routine where the N-terminal domain of each newly arriving subunit is inserted into the hydrophobic P5 pocket of the penultimate subunit, replacing the chaperone FimC and noncovalently but stably chaining the subunits to each other forming the pilus fiber [15].

FimH, the two-domain adhesin situated at the distal tip of the pilus has the ability to bind to mannose moieties with stereochemical specificity [16,17,18]. It has a pilin domain interacting with the rest of the type 1 pilus and a lectin domain that mediates host tissue binding. While the mannose-binding pocket is invariant in all sequenced UPEC isolates, diversity outside of the pocket exists with various positively selected residues playing a significant role in FimH conformational dynamics. Particularly, amino acid residue variants that lead to an extended FimH conformation interfere with infection in vivo [17]. FimH variants with positively selected residues such as A27 and V163 display an increased mannose affinity despite being attenuated in cystitis models, highlighting the importance of interchangeability between high and low mannose affinity conformations of FimH for pathogenesis in vivo [17]. This interconversion is likely needed for immune evasion such as avoiding the TLR4-mediated removal from bladder urothelium since an elongated FimH conformation may prevent the detachment from the uroplakin receptor, rendering the bacteria susceptible to TLR4-mediated clearance [19]. Sampling of multiple FimH conformations in vivo may also be an adaptation to withstand shear force, emphasizing the “catch bond” model of FimH function, where shear force exerted on the bacteria can affect the strength of interaction [20]. Furthermore, different point mutation profiles associated with the lectin domain of FimH demonstrated a favored adhesion to monomannose residues, where the same random point mutations rendered the bacteria more sensitized to the soluble inhibitors found in the oropharyngeal mucosa [21], highlighting potential adaptation mechanisms to different niches.

Type 1 pilus fibers mediate UTI progression by attachment to the urothelial surface via an interaction with the mannose moieties of the uroplakin surface of the bladder, in addition to mediating biofilm formation on biotic and abiotic surfaces as well as intracellularly [4,5,16,17,22]. Type 1 pili and FimH-expressing UPEC has the ability to invade host 5637 cells in vitro causing actin rearrangements [23]. In vivo mouse models as well as human studies revealed the requirement of type 1 piliated UPEC interaction with superficial facet cells lining the bladder to form intracellular bacterial communities (IBCs) during the first 6–18 h of infections that further can lead to chronic infections [24,25]. In vitro studies [26] also demonstrated β1 and α3 integrins (surface adhesion molecules) as key receptors for type 1 piliated UPEC. Additionally, many pathogens, including human cytomegalovirus, Group A *Streptococcus*, *Yersinia* spp. and Afa/Dr *E. coli*, gain entry into target host cells by binding integrins either directly or indirectly via matrix proteins such as fibronectin [27,28,29,30,31,32,33] The function of such integrins seem to be versatile covering cell adhesion, migration on extracellular matrix and the establishment of cell contacts in aggregates. Particularly, their role in mediating cell aggregates in breast cancer models, including highly metastatic MDA-MB-231 cells, has been investigated, and integrin α3 β1 dimers were shown to have a central role in cancer cell aggregation [34]. For instance, in solid tumors originating from epithelial cells, α3 β1 expression was increased and their inhibition with β1 antibodies was demonstrated to induce apoptosis and inhibit human breast tumors in vitro and in vivo [35].

The MDA-MB-231 cell line has been used as a model for an aggressive form of metastatic breast cancer, the triple-negative breast cancer [36]. This type of breast cancer lacks expression for both estrogen and progesterone receptors and lacks human growth factor receptor 2 (HER2) amplification [37,38,39]. In contrast to MDA-MB-231, an adenocarcinoma tumor, the MCF-7 cell line, an invasive lobular carcinoma, is non/weakly metastatic and is estrogen-responsive [40]. A WHO report indicates 685,000 breast cancer deaths occurred in 2020 alone as the leading cause of death in women [41]. While the actual death rate from breast cancer has seen a decrease from the year 1989, brought about by advances in earlier detection and treatment, high rates of mortality are a direct consequence of a readiness to metastasize [41]. Despite breast cancer being one of the most common forms of cancer encountered by women and being thought to remain at a higher incidence rate than others by 2040 [42], current therapies remain limited, leading to a need towards novel approaches to be developed in order to improve disease outcomes and quality of life.

Several studies have implicated the roles of various types of Gram-negative bacteria in interfering with tumor cell growth and viability [43]. For instance, a modified strain of *Salmonella* Typhimurium expressing a hexa-acylated lipid A was demonstrated to have antitumoral properties against CT26 colon carcinoma cells both via intravenous and intra-tumoral applications. Interestingly, intratumoral application allowed the stimulation of innate and adaptive immune responses as well as the colonization of secondary tumors. In the same colon cancer model, *E. coli* probiotics were demonstrated to have antitumoral activity, particularly via intratumoral administration, with acceptable safety profiles in vivo [44].

In this study, we hypothesized that the interaction of MDA-MB-231 and MCF-7 breast cancer cell lines with type 1 piliated UTI89 would elicit cancer cell cytotoxicity. Viability testing of both breast cancer cell lines upon incubation with type 1 piliated UPEC led to a significant cytotoxicity of tumor cells. Genetically modified UTI89 lacking type 1 adhesin *fimH* reduced the cytotoxicity on both cell lines, where *fimH* complementation rescued the cytotoxic phenotype. As mannose is a competitive inhibitor of FimH, mannose incubation of piliated UTI89 before the treatment of cancer cells reduced bacteria-mediated cytotoxicity. A further investigation of the samples revealed an attachment of statically grown, type 1 piliated UTI89 on the tumor cells compared to the vector control and shaking bacteria. Strategically engineered UPEC or UPEC products can be yet another potentially effective adjunct therapy approach to be further studied in various models.

## 2. Materials and Methods

### 2.1. Bacterial Strains

Uropathogenic *E. coli* strain UTI89, a full *fim* operon deletion mutant SJH-1106 (UTI89 Δ*fim*), *fimH* knockout in UTI89, SLC2-17-fimH, (UTI89 Δ*fimH* with pKM208) and *fimH* deletion complemented with wild-type *fimH* SLC2-33-1(UTI89 Δ*fimH*/p*fimH*) [16] were utilized for this study (courtesy of Scott J. Hultgren, Washington University in Saint Louis). Strains were kept frozen in a −80 °C freezer in 20% dimethyl sulfoxide (DMSO) (Sigma-Aldrich, St. Louis, MO, USA).

### 2.2. Bacterial Strains and Culture Conditions

Strains were streaked on nutrient agar and incubated at 37 °C overnight, where single colonies were picked the next day. Upon inoculation into a Luria Bertani/lysogeny broth (LB) (Sigma-Aldrich, St. Louis, MO, USA), 100 mL culture, all UTI89 strains were incubated under static or shaking conditions [18,45,46] at 37 °C overnight in 200 mL flasks. Sterile 100 μL LB medium was used as a vehicle control.

### 2.3. Tissue Culture Conditions

MDA-MB-231 and MCF-7 breast cancer cells were obtained from Imperial College, London UK, courtesy of Prof. Dr. Mustafa Djamgoz. Cells were grown in Dulbecco’s Modified Eagle Medium (DMEM) (Gibco by Life TechnologiesTM, Grand Island, NY, USA) supplemented with 5% fetal bovine serum (FBS) (Gibco by Life TechnologiesTM, Grand Island, NY, USA) and 2% L-glutamine (Gibco by Life TechnologiesTM, Grand Island, NY, USA). Cells were seeded into 100 mm falcon tissue culture dishes (Ultra Cruz, Dallas, TX, USA) and incubated at 37 °C, 5% CO_2_ and 100% relative humidity. They were grown to 90–100% confluence before being counted and were cultured overnight to allow them to adhere to the bottom of the wells of uncoated falcon dishes in monolayers before being used for the experiments [47].

### 2.4. Trypan Blue Exclusion Assay

A trypan blue assay (0.1%) was used to determine cell death [48]. MDA-MB-231 and MCF-7 cells were plated into 35 mm falcon tissue culture dishes (Thermo Fisher Scientific Nunc A/S, Roskilde, Denmark) with a 5 × 10^4^ cells/mL density and allowed to incubate overnight to settle before the application of the treatment. Bacterial cells were normalized to an OD_600_ of 1. One hundred microliters from the prepared suspensions was added onto the confluent layer of breast cancer cells at a multiplicity of infection (MOI) of 1000:1 [49] in 6-well plates and incubated for an hour at 37 °C, 5% CO_2_ and 100% relative humidity. Control cells received LB (vehicle control) only. Untreated breast cancer cells received only cell culture media (DMEM). Cell culture medium ± bacterial culture or control was placed with 0.1% trypan blue (Gibco by Life Technologies, Grand Island, NY, USA) in DMEM and incubated at 37 °C for 10 min. The trypan blue solution was then replaced with 1 mL of fresh culture medium, the cells were viewed at 20× magnification under an inverted microscope (Leica Microsystems Ltd. CH-9435, Heerbrug, Germany) and the percentage of alive cells ((unstained/stained) × 100) was calculated from 20 representative areas [47,48].

### 2.5. D-Mannose Treatment Protocol

A D-mannose (Now Foods, Bloomingdale, IL, USA) solution, 1% (*w*/*v*), was made by dissolving a pure powder in sterile PBS (pH 7). The bacteria were cultured in the same manner as described before. The OD_600_ of each strain was measured, and the amounts to be added to cell culture well plates were calculated. Prior to the cancer cell inoculation, static and shaking *E. coli* UTI89, UTI89 Δ*fim*, UTI89 Δ*fimH* and UTI89 Δ*fimH/pfimH* were incubated in 100 µL of 1% (*w*/*v*) D-mannose solution for 15 min at 37 °C [50].

### 2.6. Cell Surface Area and Bacterial Attachment Measurements

To obtain the cell surface area [51] and bacterial attachment measurements, all images from trypan blue assay experiments were processed via ImageJ 1.50i software (accessed on 3 July 2022) under 20× magnification. Vehicle control experiments’ breast cancer cells only received LB media, and DMEM were taken as 100% cell culture media. Cell area calculation and bacterial attachment measurements were performed from 10 representative areas for each experimental setup.

### 2.7. Hemagglutination Assay

Hemagglutination assay [52] was used to determine the expression of functional type 1 pili by different UTI89 constructs grown under different conditions. Static and shaking *E. coli* UTI89, UTI89 Δ*fim*, UTI89 Δ*fimH* and UTI89 Δ*fimH/pfimH* were grown and diluted with the growth medium to a density of 5 × 10^8^ colony-forming units/mL (cfu/mL). Equal amounts of the bacterial suspension and 1% EDTA canine erythrocyte suspension [53] in PBS were incubated in 12-well plates for 30 min at 37 °C. All incubations were performed in the presence/absence of D-mannose to reveal D-mannose sensitive agglutination. Prior to incubation with blood, equal amounts of bacterial suspensions and 1% (*w*/*v*) D-mannose were incubated for 15 min at 37 °C. The agglutination of canine erythrocytes was determined by viewing under an inverted microscope (Leica Microsystems Ltd. CH-9435, Heerbrug, Germany) at 10× magnification.

### 2.8. Statistical Analysis

All experiments were carried out 3 independent times in triplicate. Where required, Student’s *t*-test followed by a one-way ANOVA followed by a Newman–Keuls post hoc analysis was performed and results at *p* < 0.05 (*) and *p* < 0.01 (**) were considered significant. All data are represented as average ± SEM.

## 3. Results

### 3.1. Statically Grown UTI89 Exerts Cytotoxicity on Strongly and Weakly Metastatic Human Breast Cancer Cell Lines MDA-MB-231 and MCF-7

**Figure 1:**
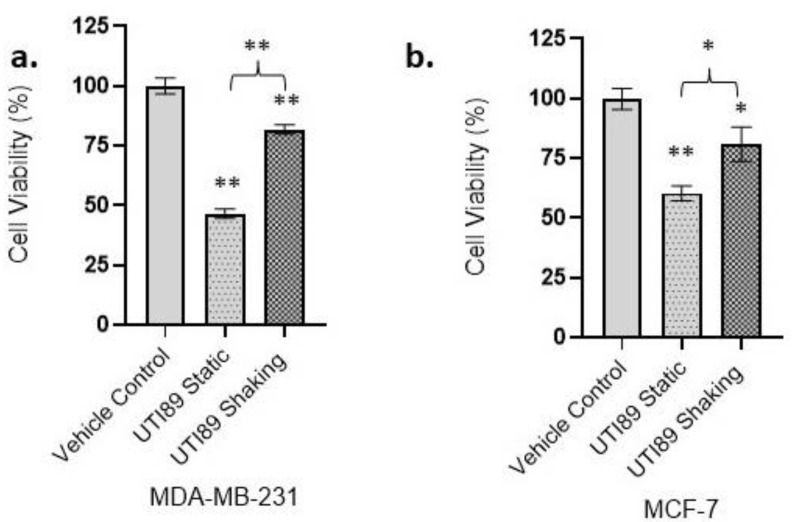
Statically grown UTI89 demonstrated significantly higher levels of cytotoxicity on highly metastatic breast cancer cells MDA-MB-231 compared to weakly metastatic breast cancer cells MCF-7. The incubation of MDA-MB-231 cells with UTI89 grown under shaking or static conditions (**a**) revealed a reduction in cell viability, with a significantly higher reduction in viability with static UTI89 (45.90% ± 1.9 viability vs. control, *p* < 0.01; *n* = 3). The incubation of MCF-7 cells with UTI89 grown under static conditions (**b**) revealed a significant reduction in cell viability (60.31% ± 3.10 viability vs. control, *p* < 0.01; *n* = 3). The reduction in cell viability was stronger for MDA-MB-231 (viability: 45.90%, *p* < 0.01 vs. control) compared to MCF-7 (viability: 60.31%, *p* < 0.01 vs. control). The incubation of MDA-MB-231 and MCF-7 cells with UTI89 grown under shaking conditions caused less reduction in cancer cell viability compared to statically grown UTI89 (MDA-MB-231: *p* < 0.01; MCF: *p* < 0.05; vs. control; *n* = 3). Data represented as ± S.E.M. (**: *p* < 0.01; *: *p* < 0.05).

Statically incubating UTI89 uropathogenic *E. coli* at 37 °C induced the expression of type 1 pili, whereas UTI89 type 1 pilus expression was reduced under shaking conditions [46] (Supplementary Figure S1 and Table S1). In order to investigate the ability of type 1 piliated UTI89 to interfere with tumor cell viability, trypan blue cytotoxicity assays were carried out. While statically grown, type 1 piliated UTI89 was shown to cause a 54.1% decrease in the viability of MDA-MB-231 cells (45.90% ± 1.9 viability compared to control, *p* < 0.01), UTI89 grown under shaking conditions exhibited only a 18.14% decrease in viability such that 81.86% ± 1.91 of the cells remained viable (Figure 1a). Statically grown UTI89 showed a 39.69% decrease in viability in MCF-7 cells (60.31% ± 3.10 viability compared to the control experiments, *p* < 0.01), while UTI89 grown under shaking conditions displayed a 19.25% reduction in cytotoxicity on the MCF-7 cell line (80.75% ± 7.17 viability compared to control (*p* < 0.05) (Figure 1b). Compared to UTI89 grown under shaking conditions, statically grown UTI89 demonstrated significantly higher levels of cytotoxicity. Overall, our results demonstrated the ability of statically grown UTI89 to cause significant levels of cytotoxicity on highly metastatic breast cancer cells MDA-MB-231 and to a lower extent, on lowly metastatic MCF-7 cells, with MDA-MB-231 appearing more susceptible to the static bacteria by 14.41% (Figure 1a,b).

In order to investigate UTI89 interactions with MCF-7 and MDA-MB-231 cells, we carried out microscopy studies. Upon investigation of cancer cells incubated with static or shaking bacterial samples or vehicle control, it was observed that the statically grown UTI89 cells were mediating contact with the MDA-MB-231 and MCF-7 cells (Table 1). While UTI89 grown under shaking conditions also demonstrated an interaction with the MDA-MB-231 and MCF-7 cells, the extent of that interaction was smaller (Table 1, Supplementary Figure S2). Cell area measurements revealed that incubations with static and shaking UTI89 also caused a significant decrease (*p* < 0.01) in MDA-MB-231 cell surface area. Statically grown bacteria and bacteria grown under shaking conditions caused a 67.47% and 63.17% decrease, respectively (Table 2, Supplementary Figure S3). On the other hand, MCF-7 incubated with static or shaking UTI89 revealed an observed increase in surface area, although that increase was not statistically significant (*p* > 0.05). A decrease in the surface area of MDA-MB-231 cells upon incubation with UTI89 may indicate an activation of apoptotic mechanisms in this highly metastatic breast cancer cell line [54].

### 3.2. UTI89-Mediated Cancer Cell Cytotoxicity Is FimH-Dependent

**Figure 2:**
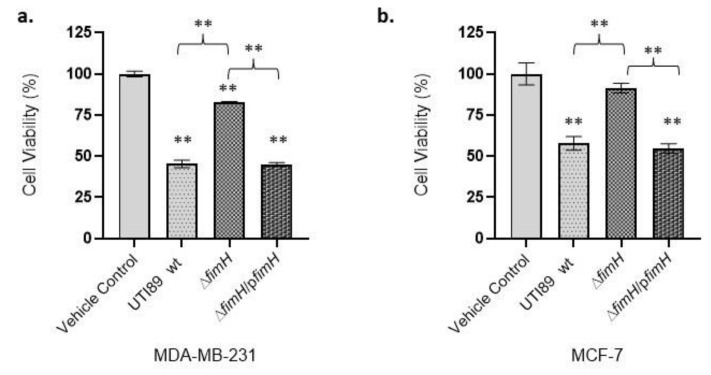
UTI89-mediated cancer cell cytotoxicity is FimH-dependent. (**a**) UTI89 Δ*fimH* exerts a reduced cytotoxicity on MDA-MB-231 cells compared to wt UTI89, leading to a higher cell viability. The cell viability is reduced back to wt levels when UTI89 Δ*fimH* is complemented with *fimH (*UTI89 Δ*fimH*/p*fimH).* (**b**) UTI89 Δ*fimH* exerts reduced cytotoxicity on MCF-7 breast cancer cells compared to wt UTI89, leading to a higher cell viability. The cell viability is reduced close to wt levels when UTI89 Δ*fimH* is complemented with *fimH (*UTI89 Δ*fimH*/p*fimH).* Data represented as ±SEM (**: *p* < 0.01; *n* = 3).

FimH adhesin is found at the tip of the type 1 pilus and allows bacterial adhesion to host tissues such as the bladder uroplakin. To investigate the role of UTI89 FimH type 1 pili adhesin in mediating the cytotoxicity of breast cancer cells, we utilized a Δ*fimH* UTI89 construct. In the absence of *fimH* (Supplementary Figure S1 and Table S1), the toxicity of UTI89 against MDA-MB-231 was significantly reduced, compared to wild-type (wt) UTI89 (*p* < 0.01, Figure 2a). After incubation of the MDA-MB-231 tumor cells with UTI89 Δ*fimH* and wt UTI89, the viability was 83.04% ± 0.29 (*p* < 0.01) and 45.39% ± 2.55 (*p* < 0.01), respectively. Upon incubation of the same bacterial strains with the weakly metastatic human breast cancer MCF-7 cell line, the cell viability was 91.35% ± 3.31 (*p* > 0.05) and 57.74% ± 4.62 (*p* < 0.01), respectively (Figure 2b). The *fimH*-complemented strain (Supplementary Figure S1 and Table S1), UTI89 Δ*fimH*/p*fimH* [16], was used to demonstrate that upon complementation of the UTI89 Δ*fimH* strain with the *fimH* gene, cancer cell viability was significantly reduced. Incubation of the MDA-MB-231 cells with the complemented strain UTI89 Δ*fimH*/p*fimH* reduced cancer cell viability to 44.97% ± 1.24 (*p* < 0.01). On the other hand, MCF-7 cells incubated with the *fimH*-complemented strain UTI89 Δ*fimH*/p*fimH* revealed a relatively higher viability value (54.79% ± 2.49, Figure 2b) compared to the MDA-MB-231 cells. These values correlate with high cytotoxicity levels of the piliated UTI89 strain grown under static conditions between the wild type and complemented strains. These results, while collectively confirming the role of FimH adhesin in the reduction of cancer cell viability, also implicate FimH in leading to cytotoxicity on human breast cancer cell lines preferentially affecting highly metastatic, estrogen-independent MDA-MB-231 cells rather than lowly metastatic, estrogen-dependent MCF-7 cells.

### 3.3. D-Mannose Prevents FimH-Mediated UTI89 Cytotoxicity of MDA-MB-231 and MCF-7 Breast Cancer Cells

**Figure 3:**
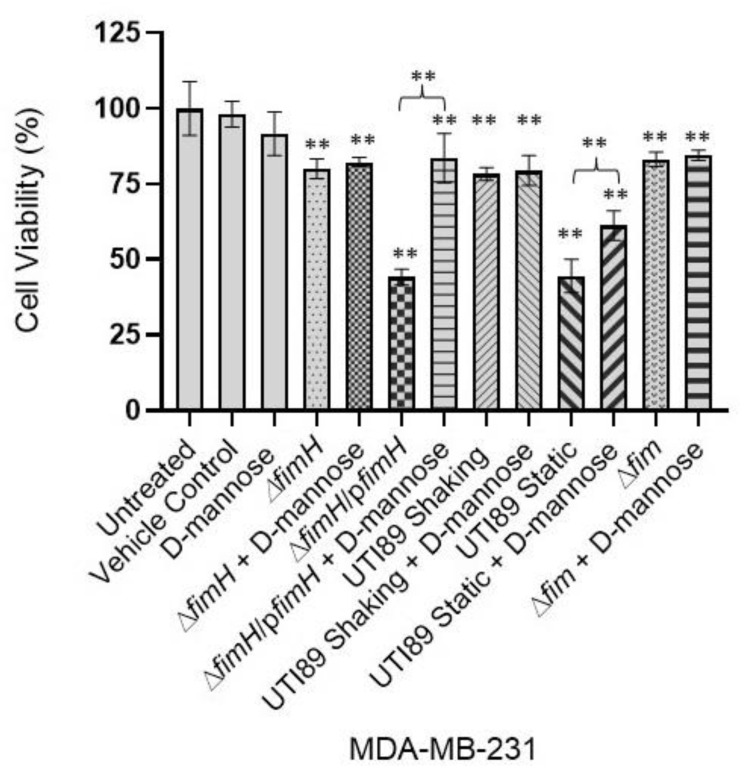
D-mannose inhibits the UTI89-mediated, FimH-dependent cytotoxicity of strongly metastatic human breast cancer cells. Various genetic constructs of UTI89 were incubated with MDA-MB-231 breast cancer cells with or without D-mannose preincubation. The constructs encoding *fimH* reduced the viability of cancer cells; however, the preincubation of these bacterial cells with D-mannose interfered with the cytotoxicity. Data represented as ±SEM (**: *p* < 0.01; *n* = 3).

**Figure 4:**
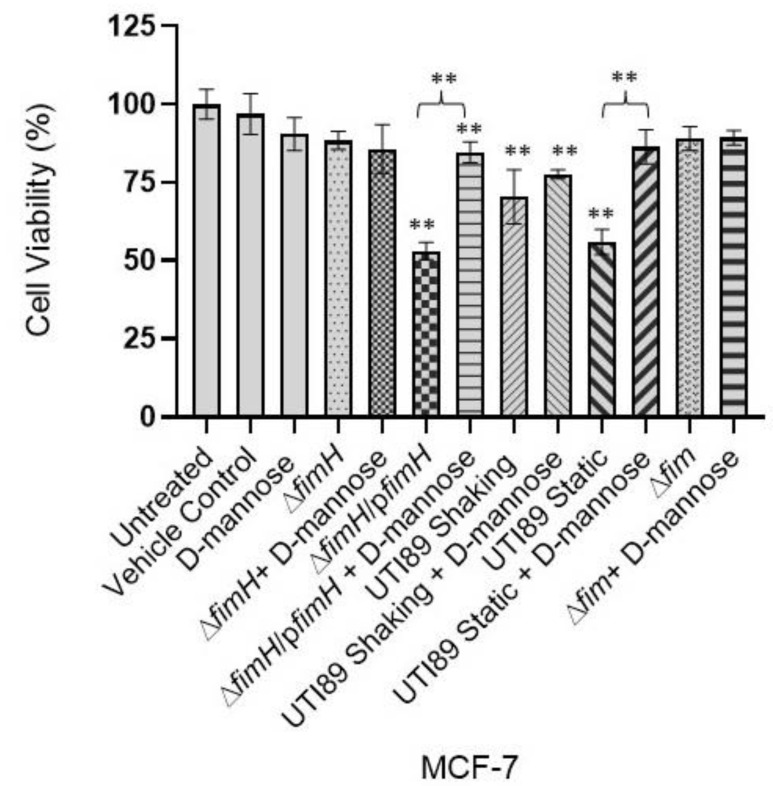
D-mannose inhibits the UTI89-mediated, FimH-dependent cytotoxicity of weakly metastatic human breast cancer cell cytotoxicity. Various genetic constructs of UTI89 were incubated with MCF-7 breast cancer cells with or without D-mannose preincubation. The constructs encoding *fimH* reduced the viability of cancer cells; however, the preincubation of these bacterial cells with D-mannose reduced the level of FimH-mediated cytotoxicity. Data represented as ±SEM (**: *p* < 0.01; *n* = 3).

Previous research has established that mannose acts as a competitive inhibitor of type 1 pilus mediated bladder adhesion and erythrocyte agglutination [16,17,18,52,53,55,56] (Supplementary Figure S1 and Table S1). We sought to investigate whether mannose could also play a role in preventing the FimH-mediated cytotoxicity exerted on the breast cancer cells. Incubating the UPEC strains expressing type 1 pili, static wt UTI89 and UTI89 Δ*fimH*/p*fimH* with D-mannose, before the MDA-MB-231 treatment, revealed a significant reduction in cytotoxicity (Figure 3). No significant change was observed when D-mannose was added to the UPEC strains that either were not grown under type 1 pili inducing conditions (shaking wt UTI89) or were genetically lacking the *fim* operon (UTI89 Δ*fim*) or *fimH (*UTI89 Δ*fimH)* (Figure 3). Similarly, a pretreatment of type 1 pili expressing bacteria (static wt UTI89 and UTI89 Δ*fimH*/p*fimH*) with D-mannose before the treatment of the weakly metastatic cancer cell line MCF-7 revealed the same trend. Preincubation of statically grown wt UTI89 and UTI89 Δ*fimH*/p*fimH* with D-mannose significantly reduced the UTI89-mediated cytotoxicity exerted on the MCF-7 human breast cancer cell line (Figure 4). However, when UTI89 Δ*fimH* or UTI89 Δ*fim* was treated with D-mannose, no significant change in the level of cytotoxicity was observed (Figure 4). These data collectively suggest that FimH mediates an interaction with the cancer cells via a mechanism similar to mannose binding, and that a functional FimH is essential for the cancer cell cytotoxicity of UTI89.

## 4. Discussion

Bacterial colonization of tumor cells has been established in various reports as a potential novel immunotherapy method against several cancers [57]. This study evaluated the significance of the role of type 1 piliated UTI89 on the cytotoxicity of MDA-MB-231 and MCF-7 cell lines. The trypan blue exclusion assay results indicated higher levels of cytotoxicity exerted by piliated UTI89 on MDA-MB-231 cells than on MCF-7, revealed by 14.41% more cell death for MDA-MB-231 cells. When grown statically, UTI89 type 1 pili expression was induced, and a growth under shaking conditions hindered their expression (Supplementary Figure S1 and Table S1). UTI89 grown under static conditions exerted significantly higher levels of cytotoxicity on both breast cancer cell lines, highlighting the role of type 1 pili in cytotoxicity. Cytotoxicity was revealed to be mediated via FimH as the *fimH* deletion exerted reduced cytotoxicity and was reversed by complementation. Furthermore, FimH is thought to bind to cancer cells in a similar mechanism to mannose binding as D-mannose interfered with type 1 pili mediated UTI89 cytotoxicity, further emphasizing the necessity of a functional FimH for cancer cell cytotoxicity.

Among other reasons, a FimH-mediated variation in cytotoxicity may be influenced by differential levels of integrins β1 and α3 expression on MDA-MB-231 cells versus MCF-7 cells [58]. Previous studies demonstrated the high levels of integrins α3 and β1 expression on an MDA-MB-231 human breast cancer cell line and lower levels of expression on MCF-7 human breast cancer cells with low metastatic potential [58,59]. Integrins were shown to contribute to tumor progression and metastasis by increasing tumor cell migration, invasion, proliferation and survival, and integrin antagonists have been considered as potential therapeutic options in several cancer types [60]. Previous work revealed the ability of type 1 piliated UPEC in mediating cell invasion in an integrin mediated manner [26]. Furthermore, the interaction between integrins α3 and β1 was shown to have been carried out via FimH [26]. β1 and α3 integrins are expressed on the highly metastatic breast cancer cell line MDA-MB-231 and they are thought to be involved in the attachment of these cells to cortical bone cells [61]. They are thought to have roles in the initiation, progression and thus metastasis of solid tumors progressing to skeletal metastases [58]. Conversely, β1 and α3 integrins are lowly expressed on the MCF-7 breast cancer cell line [59]. The more potent cytotoxic activities of type 1 piliated bacteria on integrin-expressing, estrogen-independent, strongly metastatic MDA-MB-231 cell lines compared to weakly metastatic MCF-7 cell lines may thus be due to this differential level of integrin expression. Future studies will investigate the potential involvement of integrins in FimH-mediated cancer cell cytotoxicity.

Our study demonstrates the importance of FimH in mediating tumor cell cytotoxicity. Additionally, we observed a slight nonspecific reduction in cell viability (about 10–20%) even under unpiliated conditions. This could be due to various bacterial factors including toxins encoded by UTI89 such as hly (α-hemolysin), CNF1 (cytotoxic necrotizing factor 1) and vat (vacuolating autotransporter toxin) [62,63,64]. Interestingly, UTI89 Δ*fim* led to a slight but significant D-mannose-independent cytotoxic profile on MDA-MB-231 cells which may be due to the induction of other CUP pili systems such as S-pili in the absence of the *fim* operon [55]. While the expression of these toxins or other factors under different conditions is beyond the scope of this study, future studies will investigate the involvement of these factors and others in tumor cell cytotoxicity. Furthermore, nontumoral cells, such as the nontumoral human mammary epithelial cell line MCF-10a, will be evaluated to investigate the specificity level of the observed cytotoxicity on tumor cells.

In addition to well-studied high-mannosylated uroplakin Ia glycoprotein receptors and integrins, studies indicated the ability of FimH to interact with various proteins including the kidney Tamm–Horsfall proteins [65]. FimH was also shown to bind proteins associated with the extracellular matrix including fibronectin, laminin and type IV collagens [66,67,68]. Furthermore, desmoglein 2 was revealed as a primary renal epithelial receptor for FimH [68]. The mannosylated extracellular domain of desmoglein 2 mediated an interaction with FimH in vitro, where FimH mannose binding pocket mutations such as the Q133K point mutation abolished binding [68]. Since desmoglein 2 is expressed by both MDA-MB-231 and MCF-7 cells [69] and was shown to promote breast tumor growth, where its cleavage sensitizes cells to apoptosis, future studies will investigate if the observed FimH-dependent cytotoxicity is mediated by desmoglein 2 [70].

In addition to different FimH receptors, different variants of FimH seem to affect binding affinities and specificities. For instance, while FimH variants from uropathogenic, fecal and enterohaemorrhagic *E. coli* isolates were observed to have the same binding affinity for mannose moieties, FimH from a nonuropathogenic strain O157 with the N135K mutation at the mannose-binding pocket abolished all binding [71]. It will be interesting to elucidate how different FimH variants from different pathogenic species are modulating cancer cell cytotoxicity in the context of different receptors.

Other *Enterobacteriaceae* species, such as a genetically modified *Salmonella typhimurium* strain VNP20009 used for therapeutic purposes, were demonstrated to preferentially propagate in tumors [72]. *Salmonella* spp. also utilize a variety of mechanisms such as toxin production to kill tumor cells via apoptosis or autophagy [73]. Similarly, previous work highlighted a targeting and proliferation of *E. coli* at primary and metastatic tumors in mouse models [74]. However, how this targeting happens largely remains unclear and is thought to be mediated by both passive and active mechanisms such as blood flow and chaotic vasculature formation in the tumor microenvironment pushing the bacteria towards the tumor [74]. Supporting the observations of bacterial tumor targeting, our studies also revealed a preferential accumulation of type 1 piliated bacteria around tumor cells (Table 1). Furthermore, we highlighted a molecular mechanism that implicated FimH as a factor in the targeting of bacteria toward tumor tissue to mediate cytotoxicity. To our knowledge, this is the first study demonstrating CUP-piliated bacteria as a means to mediate cancer cell viability. These data suggest that engineered UTI89 or bacterial products of UTI89 can be further studied to be potentially utilized as anticancer agents.

As synthetic biology approaches are gaining traction using engineered bacterial cells in various preclinical settings [75], FimH and CUP pili may pose a research outlet to investigate their potential action against tumor cells. Previous work established that type 1 piliated pathogenic *E. coli* induced urothelial apoptosis by suppressing NFκ-B [76]. Intratumorally injected, engineered bacteria showed promising results in clinical trials [77]. Utilizing either bacteria carrying type 1 CUP pili or isolated pili adhesin for these purposes may offer new avenues of research in future cancer studies. Before effectivity, the safety of approaches utilizing bacteria is of foremost importance, hence these studies should be carried out with animal models keeping the notion of safety as a priority. In in vivo settings, the immunostimulatory impact of bacteria in the tumor microenvironment is thought to further increase the anticancer responses [77,78,79,80,81]. Thus, two distinct yet potentially synergistic approaches of immune stimulation and FimH-mediated cancer cell cytotoxicity can be involved in antitumor mechanisms in in vivo settings.

D-mannose was shown to be protective against recurrent urinary tract infections [16,17]. *fimH*-encoding bacteria incubated in D-mannose caused a significant decrease in cancer cell cytotoxicity. Since mannose is a competitive inhibitor of FimH, bacteria likely interact with the cancer cells in a mechanism similar to that of mannose binding. This finding suggests that mannose may be utilized as a means by which the cytotoxic behavior of UPEC or UPEC products/pili can be controlled by the addition or removal of mannose.

In conclusion, our study revealed, for the first time, a breast cancer cell cytotoxicity mediated by UPEC via the CUP type 1 pili adhesin FimH. UTI89 UPEC grown under pilus-inducing, static conditions were shown to exert significantly higher levels of cytotoxicity compared to the bacteria grown under shaking conditions, where pilus biogenesis was not induced. An increased cytotoxic activity was observed on strongly metastatic MDA-MB-231 cells compared to the weakly metastatic MCF-7 cells. Cytotoxicity was effectively reduced upon deletion of the *fimH* adhesin and reversed upon complementation. The observed cytotoxic behavior can be prevented when bacteria are pretreated with D-mannose. Future studies will delineate the mechanisms behind cytotoxicity and the role of CUP pili in mediating the differential cytotoxic behavior. The knowledge generated in this study on utilizing established yet manipulable pathogens for interfering with human cancers may aid in designing microorganisms or their products as novel therapeutics and engineering bacteria/bacterial products to be studied against various cancer models.

## Figures and Tables

**Table 1:** Average number of bacteria attached per MCF-7 and MDA-MB-231 cell surface upon incubation with static or shaking UTI89. Data represented as ±SEM.

	UTI89 Static	UTI89 Shaking
MDA-MB-231	3.94 ± 0.12	2.44 ± 0.01
MCF-7	3.14 ± 0.09	2.25 ± 0.03

**Table 2:** Changes in MCF-7 and MDA-MB-231 breast cancer cell % surface area upon incubation with static or shaking UTI89. Data represented as ±SEM.

	Vehicle Control	UTI89 Static	UTI89 Shaking
MDA-MB-231	100 ± 8.95	32.5 ± 2.4	36.8 ± 3.69
MCF-7	100 ± 13.45	153.35 ± 21.13	156.78 ± 23.91

## Data Availability

Not applicable.

## Supplementary Materials

The following supporting information can be downloaded at: https://www.mdpi.com/article/10.3390/pathogens12060751/s1, Figure S1: Light microscopy images of E. coli UTI89 grown under static or shaking conditions, UTI89 *Δfim*, UTI89 *ΔfimH* and UTI89 *ΔfimH/pfimH* incubated with canine blood with/without D-mannose (10× magnification); Table S1: Red Blood Cell Hemagglutination of various constructs of UTI89 with/without D-mannose; Figure S2: Inverted light microscopy images revealed that UTI89 grown statically has increased interaction with both MDA-MB-231 and MCF-7 breast cancer cells compared to the UTI89 grown under shaking conditions (×20 magnification; arrows point to the enlarged area); Figure S3: Inverted light microscopy images revealed significant cell surface area reduction in MDA-MB-231 cells upon incubation with both static and shaking UTI89 compared to MCF-7 breast cancer cells (×20 magnification).

## Sample Availability

Samples are available from the authors.

## References

[1] Hooton T.M., Stamm W.E. (1997). Diagnosis and treatment of uncomplicated urinary tract infection. Infect. Dis. Clin. N. Am..

[2] Svanborg C., Godaly G. (1997). Bacterial virulence in urinary tract infection. Infect. Dis. Clin. N. Am..

[3] Connell I., Agace W., Klemm P., Schembri M., Mărild S., Svanborg C. (1996). Type 1 fimbrial expression enhances *Escherichia coli* virulence for the urinary tract. Proc. Natl. Acad. Sci. USA.

[4] Mulvey M.A., Lopez-Boado Y.S., Wilson C.L., Roth R., Parks W.C., Heuser J., Hultgren S.J. (1998). Induction and evasion of host defenses by type 1-piliated uropathogenic *Escherichia coli*. Science.

[5] Anderson G.G., Palermo J.J., Schilling J.D., Roth R., Heuser J., Hultgren S.J. (2003). Intracellular bacterial biofilm-like pods in urinary tract infections. Science.

[6] Melican K., Sandoval R.M., Kader A., Josefsson L., Tanner G.A., Molitoris B.A., Richter-Dahlfors A. (2011). Uropathogenic *Escherichia coli* P and Type 1 fimbriae act in synergy in a living host to facilitate renal colonization leading to nephron obstruction. PLoS Pathog..

[7] Hultgren S.J., Porter T.N., Schaeffer A.J., Duncan J.L. (1985). Role of type 1 pili and effects of phase variation on lower urinary tract infections produced by *Escherichia coli*. Infect. Immun..

[8] Wright K.J., Seed P.C., Hultgren S.J. (2007). Development of intracellular bacterial communities of uropathogenic *Escherichia coli* depends on type 1 pili. Cell Microbiol..

[9] Volkan E., Kalas V., Hultgren S., Tang Y.-W., Sussman M., Liu D., Poxton I., Schwartzman J. (2015). Molecular Medical Microbiology (Second Edition), Chapter 8-Pili and Fimbriae of Gram-Negative Bacteria.

[10] Volkan E., Ford B.A., Pinkner J.S., Dodson K.W., Henderson N.S., Thanassi D.G., Waksman G., Hultgren S.J. (2012). Domain activities of PapC usher reveal the mechanism of action of an *Escherichia coli* molecular machine. Proc. Natl. Acad Sci. USA.

[11] Sauer F.G., Fütterer K., Pinkner J.S., Dodson K.W., Hultgren S.J., Waksman G. (1999). Structural basis of chaperone function and pilus biogenesis. Science.

[12] Spaulding C.N., Hultgren S.J. (2016). Adhesive Pili in UTI Pathogenesis and Drug Development. Pathogens.

[13] Phan G., Remaut H., Wang T., Allen W.J., Pirker K.F., Lebedev A., Henderson N.S., Geibel S., Volkan E., Yan J. (2011). Crystal structure of the FimD usher bound to its cognate FimC-FimH substrate. Nature.

[14] Volkan E., Kalas V., Pinkner J.S., Dodson K.W., Henderson N.S., Pham T., Waksman G., Delcour A.H., Thanassi D.G., Hultgren S.J. (2013). Molecular basis of usher pore gating in *Escherichia coli* pilus biogenesis. Proc. Natl. Acad. Sci. USA.

[15] Ford B., Verger D., Dodson K., Volkan E., Kostakioti M., Elam J., Pinkner J., Waksman G., Hultgren S. (2012). The structure of the PapD-PapGII pilin complex reveals an open and flexible P5 pocket. J. Bacteriol..

[16] Chen S.L., Hung C.S., Pinkner J.S., Walker J.N., Cusumano C.K., Li Z., Bouckaert J., Gordon J.I., Hultgren S.J. (2009). Positive selection identifies an in vivo role for FimH during urinary tract infection in addition to mannose binding. Proc. Natl. Acad. Sci. USA.

[17] Schwartz D.J., Kalas V., Pinkner J.S., Chen S.L., Spaulding C.N., Dodson K.W., Hultgren S.J. (2013). Positively selected FimH residues enhance virulence during urinary tract infection by altering FimH conformation. Proc. Natl. Acad. Sci. USA.

[18] Greene S.E., Hibbing M.E., Janetka J., Chen S.L., Hultgren S.J. (2015). Human Urine Decreases Function and Expression of Type 1 Pili in Uropathogenic *Escherichia coli*. mBio.

[19] Schilling J.D., Martin S.M., Hung C.S., Lorenz R.G., Hultgren S.J. (2003). Toll-like receptor 4 on stromal and hematopoietic cells mediates innate resistance to uropathogenic *Escherichia coli*. Proc. Natl. Acad. Sci. USA.

[20] Sokurenko E.V., Vogel V., Thomas W.E. (2008). Catch-bond mechanism of force-enhanced adhesion: Counterintuitive, elusive, but... widespread?. Cell Host Microbe.

[21] Sokurenko E.V., Chesnokova V., Dykhuizen D.E., Ofek I., Wu X.R., Krogfelt K.A., Struve C., Schembri M.A., Hasty D.L. (1998). Pathogenic adaptation of *Escherichia coli* by natural variation of the FimH adhesin. Proc. Natl. Acad. Sci. USA.

[22] Wu X.R., Sun T.T., Medina J.J. (1996). In vitro binding of type 1-fimbriated *Escherichia coli* to uroplakins Ia and Ib: Relation to urinary tract infections. Proc. Natl. Acad. Sci. USA.

[23] Martinez J.J., Mulvey M.A., Schilling J.D., Pinkner J.S., Hultgren S.J. (2000). Type 1 pilus-mediated bacterial invasion of bladder epithelial cells. EMBO J..

[24] Spaulding C.N., Schreiber HL 4th Zheng W., Dodson K.W., Hazen J.E., Conover M.S., Wang F., Svenmarker P., Luna-Rico A., Francetic O., Andersson M. (2018). Functional role of the type 1 pilus rod structure in mediating host-pathogen interactions. Elife.

[25] Bishop B.L., Duncan M.J., Song J., Li G., Zaas D., Abraham S.N. (2007). Cyclic AMP-regulated exocytosis of *Escherichia coli* from infected bladder epithelial cells. Nat. Med..

[26] Eto D.S., Jones T.A., Sundsbak J.L., Mulvey M.A. (2007). Integrin-mediated host cell invasion by type 1-piliated uropathogenic *Escherichia coli*. PLoS Pathog..

[27] Guignot J., Bernet-Camard M.F., Poüs C., Plançon L., Le Bouguenec C., Servin A.L. (2001). Polarized entry of uropathogenic Afa/Dr diffusely adhering *Escherichia coli* strain IH11128 into human epithelial cells: Evidence for alpha5beta1 integrin recognition and subsequent internalization through a pathway involving caveolae and dynamic unstable microtubules. Infect. Immun..

[28] Scibelli A., Roperto S., Manna L., Pavone L.M., Tafuri S., Della Morte R., Staiano N. (2007). Engagement of integrins as a cellular route of invasion by bacterial pathogens. Vet. J..

[29] Wang X., Huang D.Y., Huong S.M., Huang E.S. (2005). Integrin alphavbeta3 is a coreceptor for human cytomegalovirus. Nat. Med..

[30] Cue D., Lam H., Cleary P.P. (2001). Genetic dissection of the *Streptococcus pyogenes* M1 protein: Regions involved in fibronectin binding and intracellular invasion. Microb. Pathog..

[31] Isberg R.R., Leong J.M. (1990). Multiple beta 1 chain integrins are receptors for invasin, a protein that promotes bacterial penetration into mammalian cells. Cell.

[32] Tran Van Nhieu G., Isberg R.R. (1993). Bacterial internalization mediated by beta 1 chain integrins is determined by ligand affinity and receptor density. EMBO J..

[33] Plançon L., Du Merle L., Le Friec S., Gounon P., Jouve M., Guignot J., Servin A., Le Bouguénec C. (2003). Recognition of the cellular beta1-chain integrin by the bacterial AfaD invasin is implicated in the internalization of afa-expressing pathogenic *Escherichia coli* strains. Cell Microbiol..

[34] Lusche D.F., Klemme M.R., Soll B.A., Reis R.J., Forrest C.C., Nop T.S., Wessels D.J., Berger B., Glover R., Soll D.R. (2019). Integrin α-3 ß-1′s central role in breast cancer, melanoma and glioblastoma cell aggregation revealed by antibodies with blocking activity. MAbs.

[35] Desgrosellier J.S., Cheresh D.A. (2010). Integrins in cancer: Biological implications and therapeutic opportunities. Nat. Rev. Cancer.

[36] Hero T., Bühler H., Kouam P.N., Priesch-Grzeszowiak B., Lateit T., Adamietz I.A. (2019). The Triple-negative Breast Cancer Cell Line MDA-MB 231 Is Specifically Inhibited by the Ionophore Salinomycin. Anticancer Res..

[37] Perou C.M., Sørlie T., Eisen M.B., van de Rijn M., Jeffrey S.S., Rees C.A., Pollack J.R., Ross D.T., Johnsen H., Akslen L.A. (2000). Molecular portraits of human breast tumours. Nature.

[38] Lee A., Djamgoz M.B.A. (2018). Triple negative breast cancer: Emerging therapeutic modalities and novel combination therapies. Cancer Treat. Rev..

[39] Zhu Z., Boobis A.R., Edwards R.J. (2008). Identification of estrogen-responsive proteins in MCF-7 human breast cancer cells using label-free quantitative proteomics. Proteomics.

[40] Chen K., Satlof L., Stoffels G., Kothapalli U., Ziluck N., Lema M., Poretsky L., Avtanski D. (2019). Cytokine secretion in breast cancer cells-MILLIPLEX assay data. Data Brief..

[41] Sung H., Ferlay J., Siegel R.L., Laversanne M., Soerjomataram I., Jemal A., Bray F. (2021). Global cancer statistics 2020: GLOBOCAN estimates of incidence and mortality worldwide for 36 cancers in 185 coun-tries. CA Cancer J. Clin..

[42] Rahib L., Wehner M.R., Matrisian L.M., Nead K.T. (2021). Estimated Projection of US Cancer Incidence and Death to 2040. JAMA Netw. Open.

[43] Lee C.H., Wu C.L., Shiau A.L. (2008). Salmonella choleraesuis as an anticancer agent in a syngeneic model of orthotopic hepatocellular carcinoma. Int. J. Cancer.

[44] Kocijancic D., Felgner S., Schauer T., Frahm M., Heise U., Zimmermann K., Erhardt M., Weiss S. (2017). Local application of bacteria improves safety of Salmonella -mediated tumor therapy and retains advantages of systemic infection. Oncotarget.

[45] Floyd R.V., Upton M., Hultgren S.J., Wray S., Burdyga T.V., Winstanley C. (2012). *Escherichia coli*-mediated impairment of ureteric contractility is uropathogenic *E. coli* specific. J. Infect. Dis..

[46] Hultgren S.J., Schwan W.R., Schaeffer A.J., Duncan J.L. (1986). Regulation of production of type 1 pili among urinary tract isolates of *Escherichia coli*. Infect. Immun..

[47] Isbilen O., Volkan E. (2021). Allium willeanum Holmboe exerts anticancer activities on metastatic breast cancer cells MCF-7 and MDA-MB-231. Heliyon.

[48] Rizaner N., Uzun S., Fraser S.P., Djamgoz M.B.A., Altun S. (2020). Riluzole: Anti-invasive effects on rat prostate cancer cells under normoxic and hypoxic conditions. Basic Clin. Pharmacol. Toxicol..

[49] Rubinstein M.R., Baik J.E., Lagana S.M., Han R.P., Raab W.J., Sahoo D., Dalerba P., Wang T.C., Han Y.W. (2019). *Fusobacterium nucleatum* promotes colorectal cancer by inducing Wnt/β-catenin modulator Annexin A1. EMBO Rep..

[50] Spaulding C.N., Klein R.D., Ruer S., Kau A.L., Schreiber H.L., Cusumano Z.T., Dodson K.W., Pinkner J.S., Fremont D.H., Janetka J.W. (2017). Selective depletion of uropathogenic *E. coli* from the gut by a FimH antagonist. Nature.

[51] Pasqualato A., Lei V., Cucina A., Dinicola S., D’Anselmi F., Proietti S., Masiello M.G., Palombo A., Bizzarri M. (2013). Shape in migration: Quantitative image analysis of migrating chemoresistant HCT-8 colon cancer cells. Cell Adhes. Migr..

[52] Johnson J.R., Brown J.J., Ahmed P. (1998). Diversity of hemagglutination phenotypes among P-fimbriated wild-type strains of *Escherichia coli* in relation to papG allele repertoire. Clin. Diagn. Lab. Immunol..

[53] Senior D.F., deMan P., Svanborg C. (1992). Serotype, hemolysin production, and adherence characteristics of strains of *Escherichia coli* causing urinary tract infection in dogs. Am. J. Vet. Res..

[54] Bortner C., Cidlowski J. (2002). Apoptotic volume decrease and the incredible shrinking cell. Cell Death Differ..

[55] Greene S.E., Pinkner J.S., Chorell E., Dodson K.W., Shaffer C.L., Conover M.S., Livny J., Hadjifrangiskou M., Almqvist F., Hultgren S.J. (2014). Pilicide ec240 disrupts virulence circuits in uropathogenic *Escherichia coli*. mBio.

[56] Lenger S.M., Bradley M.S., Thomas D.A., Bertolet M.H., Lowder J.L., Sutcliffe S. (2020). D-mannose vs other agents for recurrent urinary tract infection prevention in adult women: A systematic review and meta-analysis. Am. J. Obstet. Gynecol..

[57] Duong M.T.Q., Qin Y., You S.H., Min J.J. (2019). Bacteria-cancer interactions: Bacteria-based cancer therapy. Exp. Mol. Med..

[58] Lundström A., Holmbom J., Lindqvist C., Nordström T. (1998). The role of alpha2 beta1 and alpha3 beta1 integrin receptors in the initial anchoring of MDA-MB-231 human breast cancer cells to cortical bone matrix. Biochem. Biophys. Res. Commun..

[59] Taherian A., Li X., Liu Y., Haas T.A. (2011). Differences in integrin expression and signaling within human breast cancer cells. BMC Cancer.

[60] van der Pluijm, Vloedgraven H., Papapoulos S., Löwick C., Grzesik W., Kerr J., Robey P.G. (1997). Attachment characteristics and involvement of integrins in adhesion of breast cancer cell lines to extracellular bone matrix components. Lab. Investig..

[61] Pantano F., Croset M., Driouch K., Bednarz-Knoll N., Iuliani M., Ribelli G., Bonnelye E., Wikman H., Geraci S., Bonin F. (2021). Integrin alpha5 in human breast cancer is a mediator of bone metastasis and a therapeutic target for the treatment of osteolytic lesions. Oncogene.

[62] Wiles T.J., Kulesus R.R., Mulvey M.A. (2008). Origins and virulence mechanisms of uropathogenic *Escherichia coli*. Exp. Mol. Pathol..

[63] Garcia T.A., Ventura C.L., Smith M.A., Merrell D.S., O’Brien A.D. (2013). Cytotoxic necrotizing factor 1 and hemolysin from uropathogenic *Escherichia coli* elicit different host responses in the murine bladder. Infect. Immun..

[64] Subashchandrabose S., Mobley H.L.T. (2015). Virulence and Fitness Determinants of Uropathogenic *Escherichia coli*. Microbiol. Spectr..

[65] Bates J.M., Raffi H.M., Prasadan K., Mascarenhas R., Laszik Z., Maeda N., Hultgren S.J., Kumar S. (2004). Tamm-Horsfall protein knockout mice are more prone to urinary tract infection: Rapid communication. Kidney Int..

[66] Kukkonen M., Raunio T., Virkola R., Lähteenmäki K., Mäkelä P.H., Klemm P., Clegg S., Korhonen T.K. (1993). Basement membrane carbohydrate as a target for bacterial adhesion: Binding of type I fimbriae of *Salmonella enterica* and *Escherichia coli* to laminin. Mol. Microbiol..

[67] Bouckaert J., Mackenzie J., de Paz J.L., Chipwaza B., Choudhury D., Zavialov A., Mannerstedt K., Anderson J., Piérard D., Wyns L. (2006). The affinity of the FimH fimbrial adhesin is receptor-driven and quasi-independent of *Escherichia coli* pathotypes. Mol. Microbiol..

[68] McLellan L.K., McAllaster M.R., Kim A.S., Tóthová L’., Olson P.D., Pinkner J.S., Daugherty A.L., Hreha T.N., Janetka J.W., Fremont D.H. (2021). A host receptor enables type 1 pilus-mediated pathogenesis of *Escherichia coli* pyelonephritis. PLoS Pathog..

[69] Davies E., Cochrane R., Hiscox S., Jiang W., Sweetland H., Mansel R. (1997). The role of desmoglein 2 and E-cadherin in the invasion and motility of human breast cancer cells. Int. J. Oncol..

[70] Chang P.H., Chen M.C., Tsai Y.P., Tan G.Y.T., Hsu P.H., Jeng Y.M., Tsai Y.F., Yang M.H., Hwang-Verslues W.W. (2021). Interplay between desmoglein2 and hypoxia controls metastasis in breast cancer. Proc. Natl. Acad. Sci. USA.

[71] Shaikh N., Holt N.J., Johnson J.R., Tarr P.I. (2007). Fim operon variation in the emergence of Enterohemorrhagic *Escherichia coli*: An evolutionary and functional analysis. FEMS Microbiol. Lett..

[72] Clairmont C., Lee K.C., Pike J., Ittensohn M., Low K.B., Pawelek J., Bermudes D., Brecher S.M., Margitich D., Turnier J. (2000). Biodistribution and genetic stability of the novel antitumor agent VNP20009, a genetically modified strain of *Salmonella typhimurium*. J. Infect. Dis..

[73] Ganai S., Arenas R.B., Sauer J.P., Bentley B., Forbes N.S. (2011). In tumors *Salmonella* migrate away from vasculature toward the transition zone and induce apoptosis. Cancer Gene Ther..

[74] Min J.J., Kim H.J., Park J.H., Moon S., Jeong J.H., Hong Y.J., Cho K.O., Nam J.H., Kim N., Park Y.K. (2008). Noninvasive real-time imaging of tumors and metastases using tumor-targeting light-emitting *Escherichia coli*. Mol. Imaging Biol..

[75] Kang M., Choe D., Kim K., Cho B.K., Cho S. (2020). Synthetic Biology Approaches in The Development of Engineered Therapeutic Microbes. Int. J. Mol. Sci..

[76] Klumpp D.J., Weiser A.C., Sengupta S., Forrestal S.G., Batler R.A., Schaeffer A.J. (2001). Uropathogenic *Escherichia coli* potentiates type 1 pilus-induced apoptosis by suppressing NF-kappaB. Infect. Immun..

[77] Zhou S., Gravekamp C., Bermudes D., Liu K. (2018). Tumour-targeting bacteria engineered to fight cancer. Nat. Rev. Cancer.

[78] Mi Z., Feng Z.C., Li C., Yang X., Ma M.T., Rong P.F. (2019). Salmonella-Mediated Cancer Therapy: An Innovative Therapeutic Strategy. J. Cancer.

[79] McCarthy E.F. (2006). The toxins of William B. Coley and the treatment of bone and soft-tissue sarcomas. Iowa Orthop. J..

[80] Volkan E., Isbilen O. (2018). Microbiological approaches to Treating Cancer Cancer Therapy.

[81] Hoption Cann S.A., van Netten J.P., van Netten C. (2003). Dr William Coley and tumour regression: A place in history or in the future. Postgrad. Med. J..

