# Supplementary material for: FimH and Type 1 Pili Mediated Tumor Cell Cytotoxicity by Uropathogenic Escherichia coli In Vitro
Source: Pathogens. 2023 May 23;12(6):751. doi: 10.3390/pathogens12060751 (PMC10304368; doi:10.3390/pathogens12060751)

Figure S1. Light microscopy images of E. coli UTI89 grown under static or shaking conditions, UTI89 *Δfim*, UTI89 *ΔfimH* and UTI89 *ΔfimH/pfimH* incubated with canine blood with/without D-mannose (10× magnification).

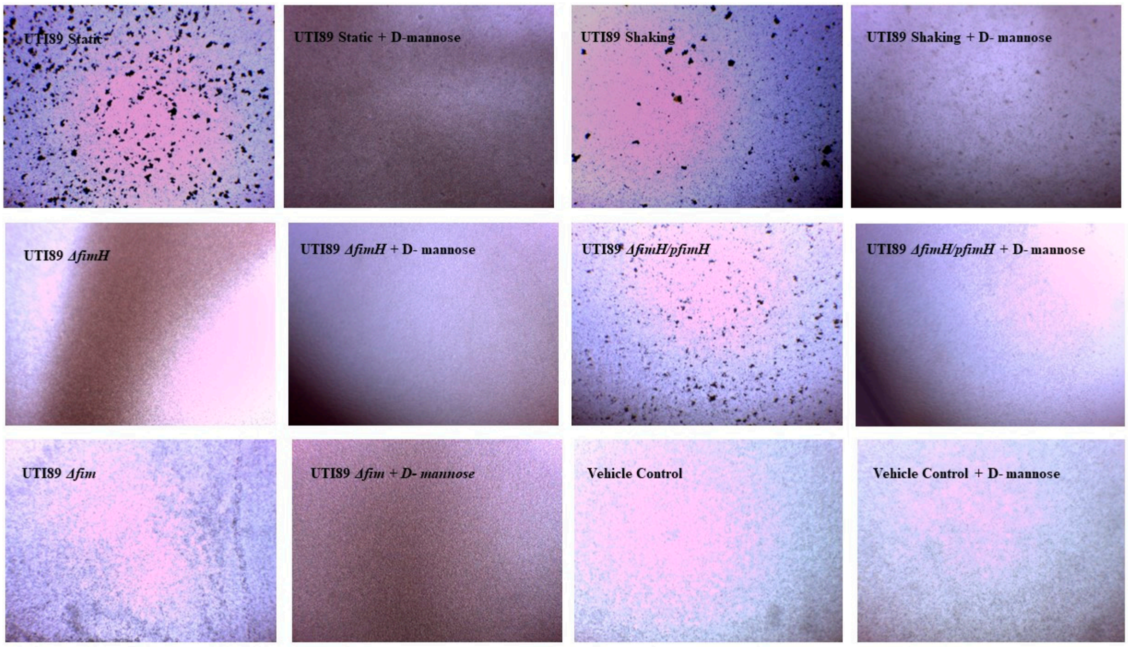

Table S1. Red Blood Cell Hemagglutination of various constructs of UTI89 with/without D-mannose.

	**UTI89 Static**	**UTI89 Shaking**	**UTI89 *ΔfimH***	**UTI89 *ΔfimH/pfimH***	**UTI89 *Δfim***
**Mannose (–)**	+++	+	–	++	–
**Mannose (+)**	–	–	–	–	–

"–": No agglutination "+": Low agglutination "++": Moderate agglutination "+++": High agglutination.

Figure S2. Inverted light microscopy images revealed that UTI89 grown statically has increased interaction with both MDA-MB-231 and MCF-7 breast cancer cells compared to the UTI89 grown under shaking conditions (×20 magnification; arrows point to the enlarged area).

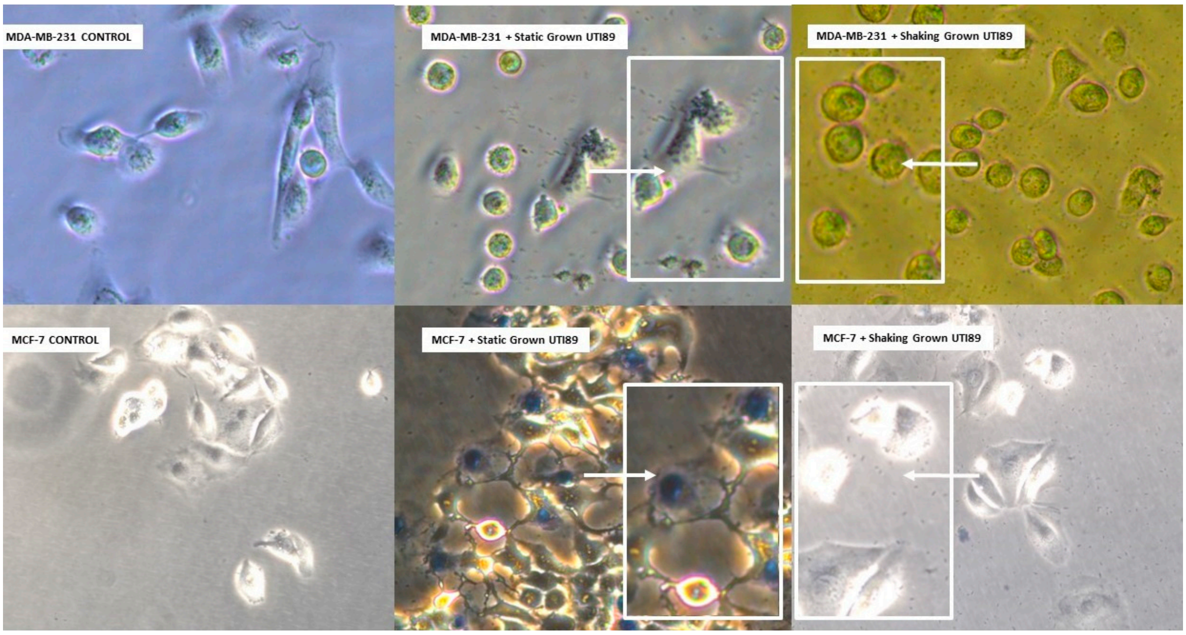

Figure S3. Inverted light microscopy images revealed significant cell surface area reduction in MDA-MB-231 cells upon incubation with both static and shaking UTI89 compared to MCF-7 breast cancer cells (×20 magnification).

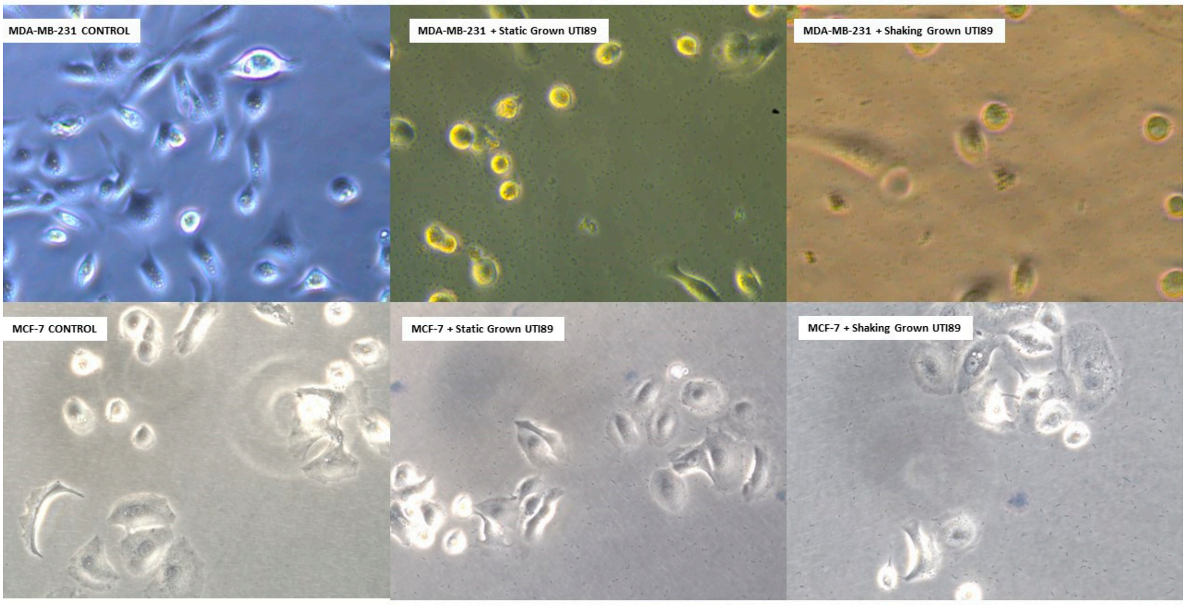

Supplement: Supplementary file 1 [file pathogens-12-00751-s001.zip › pathogens-2368460-supplementary.pdf]
